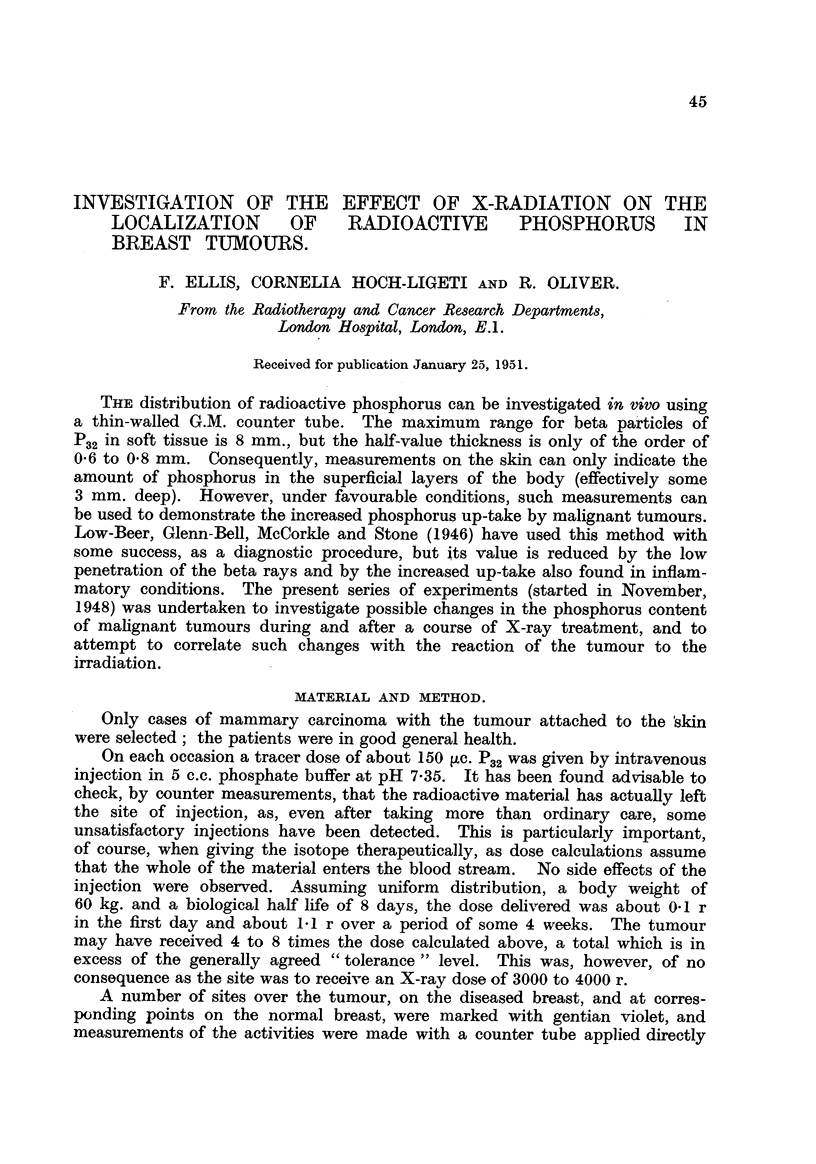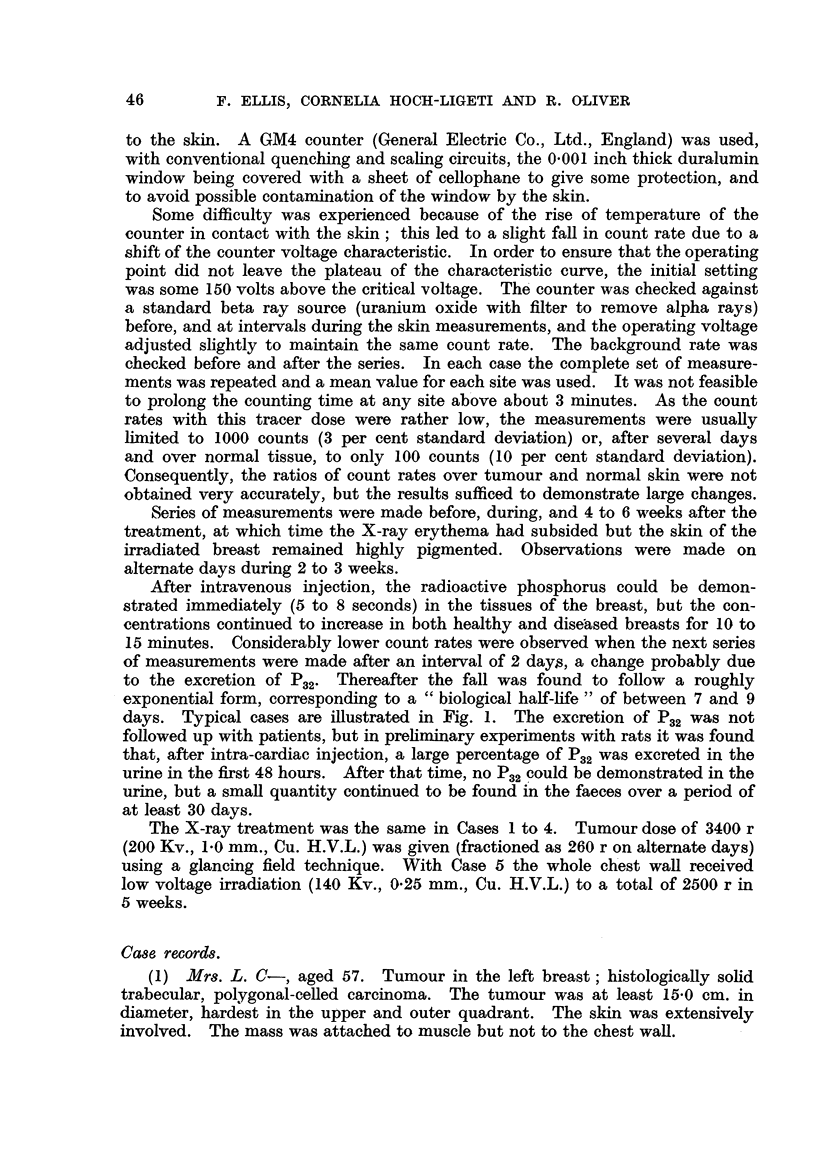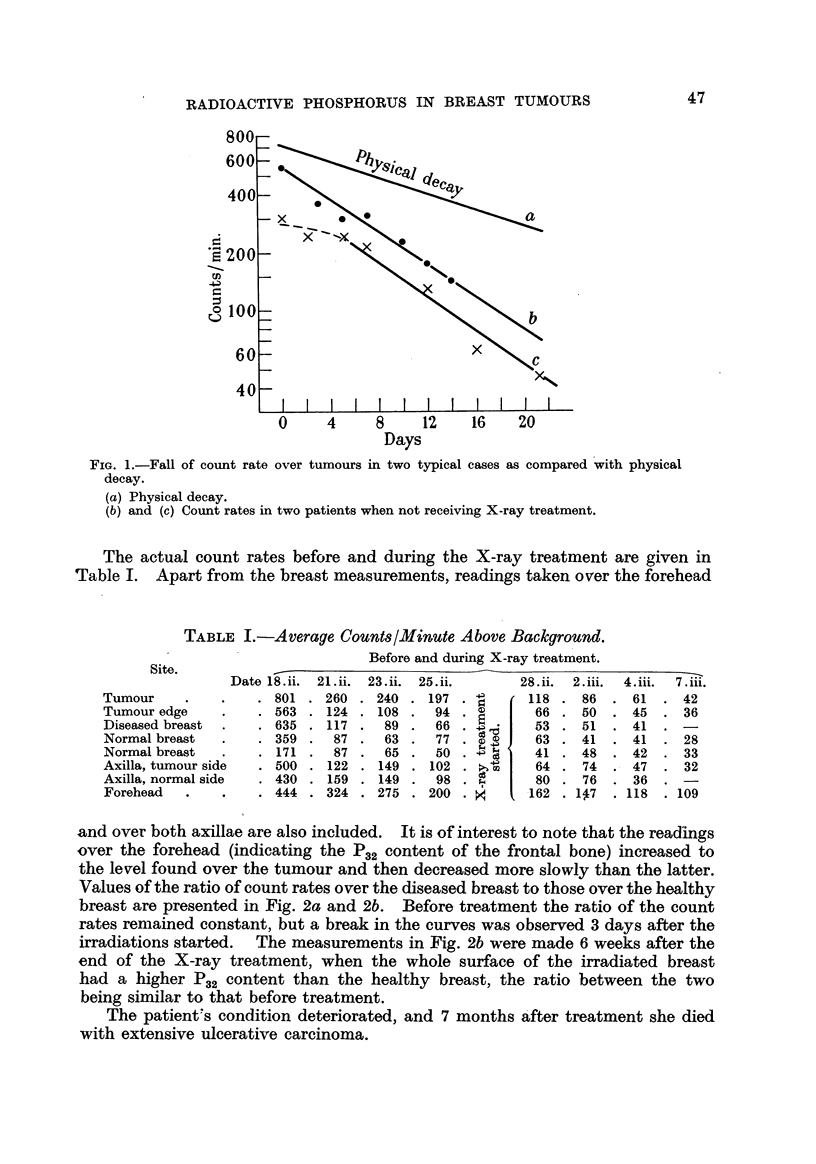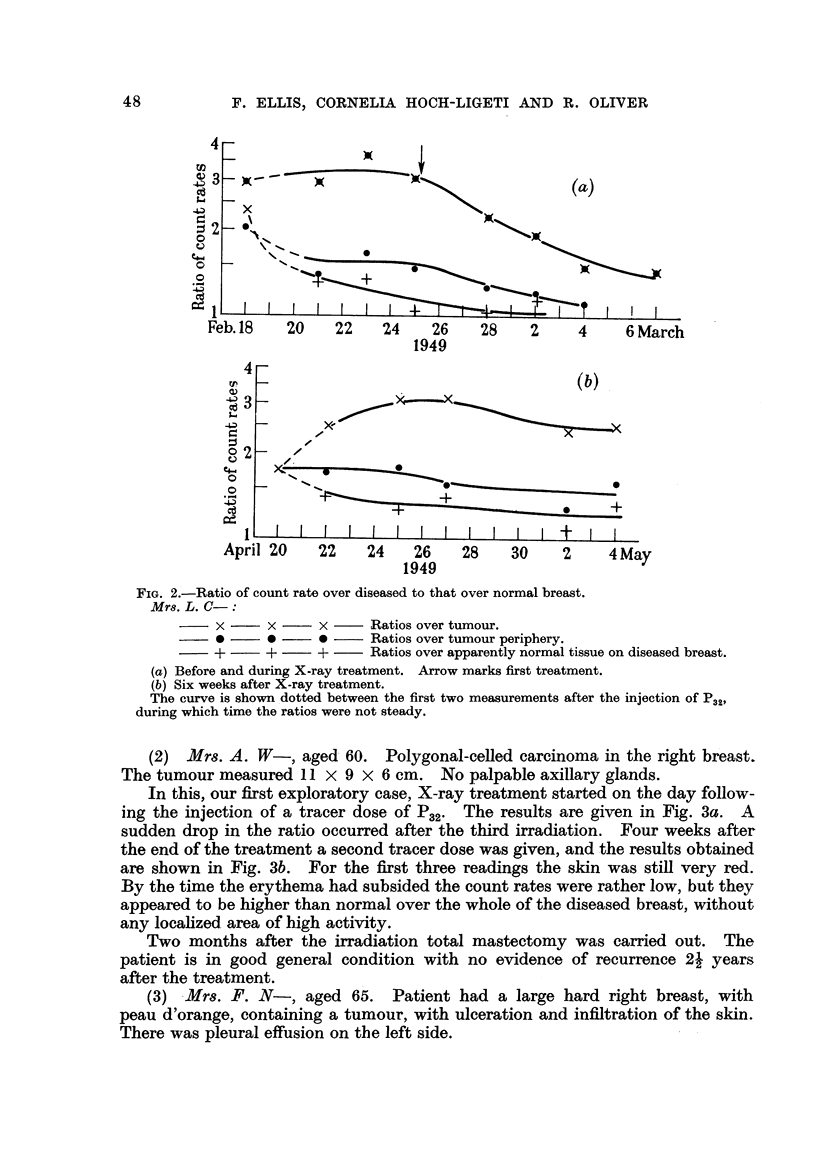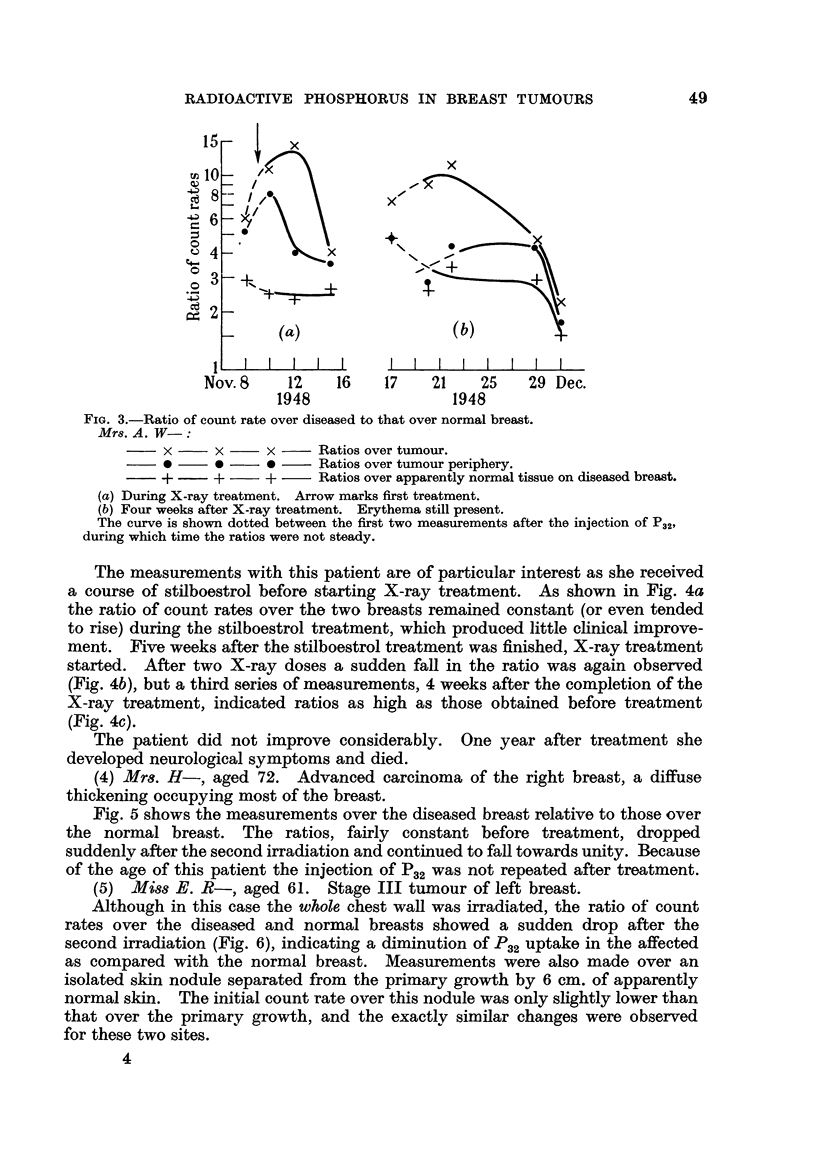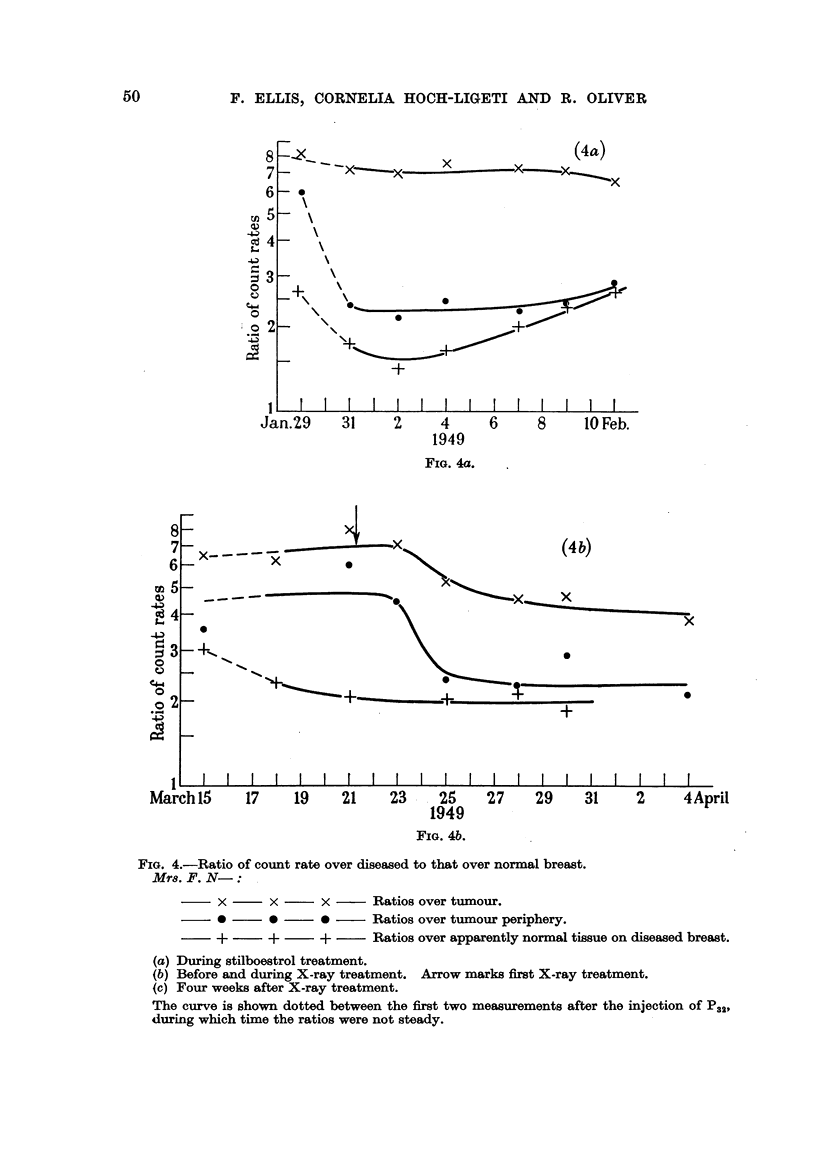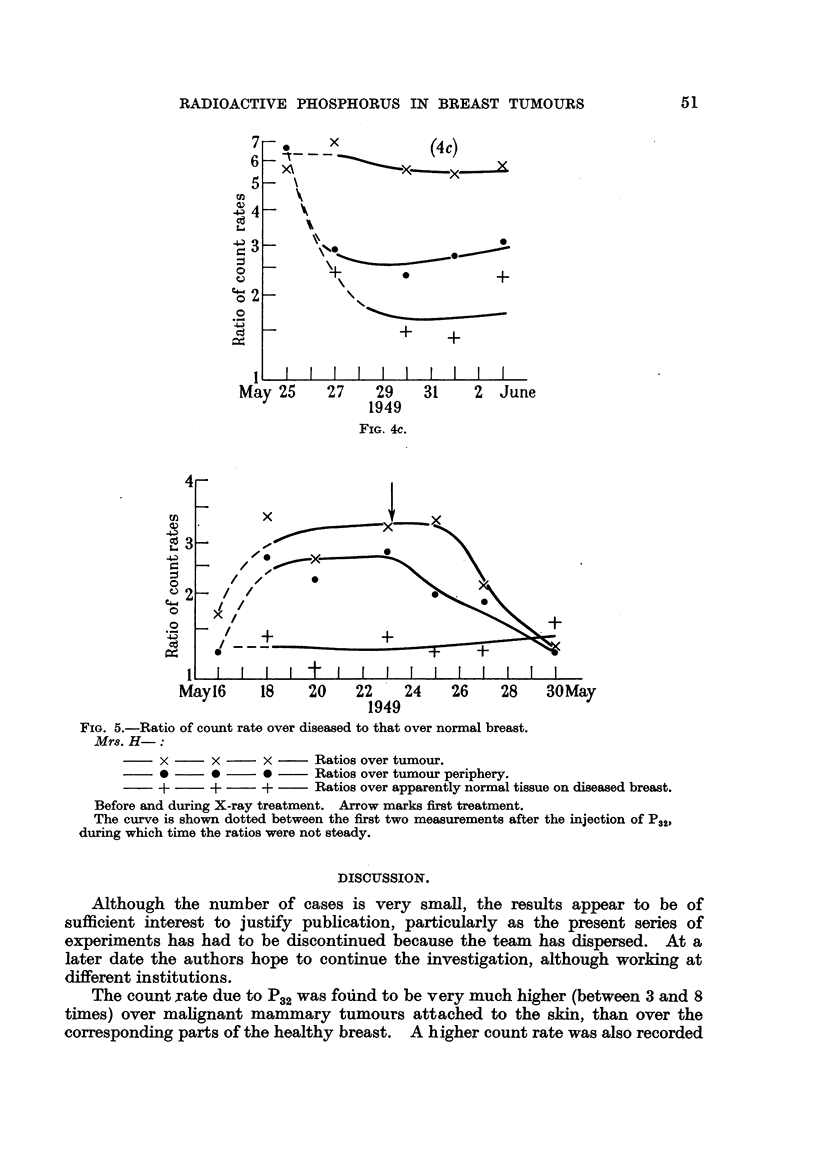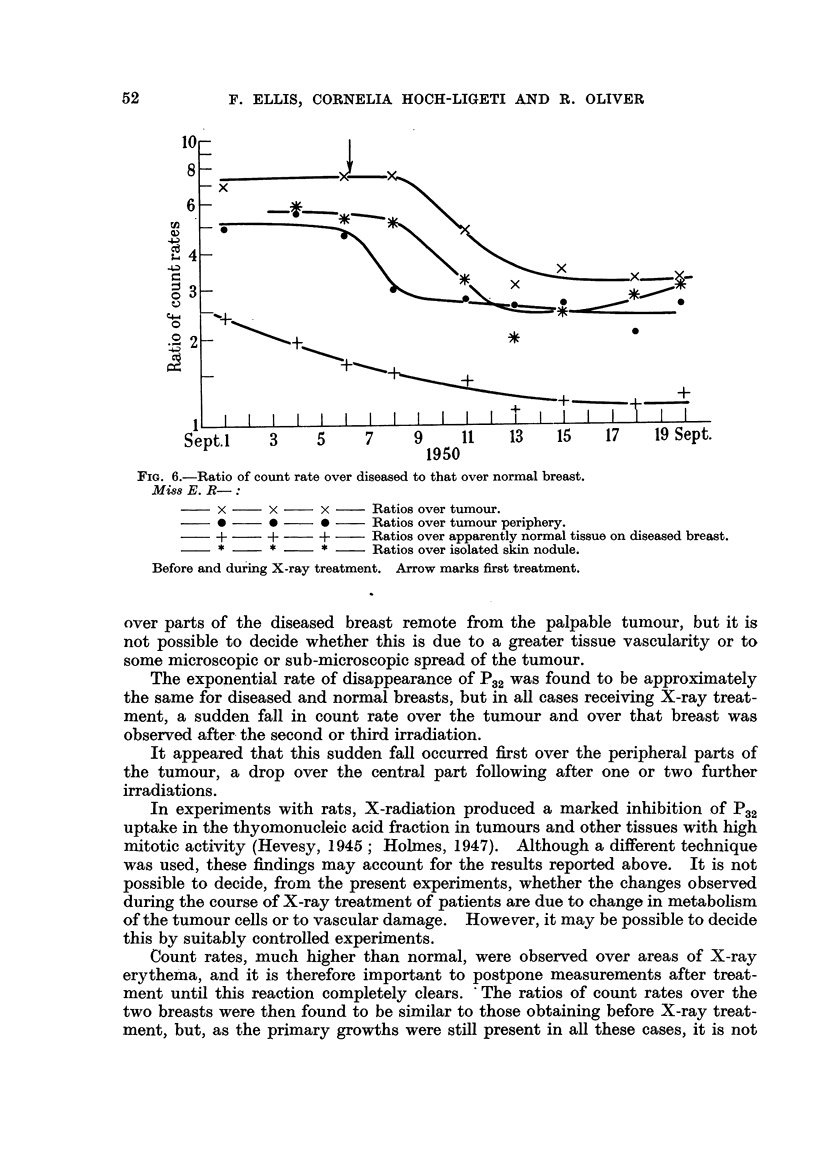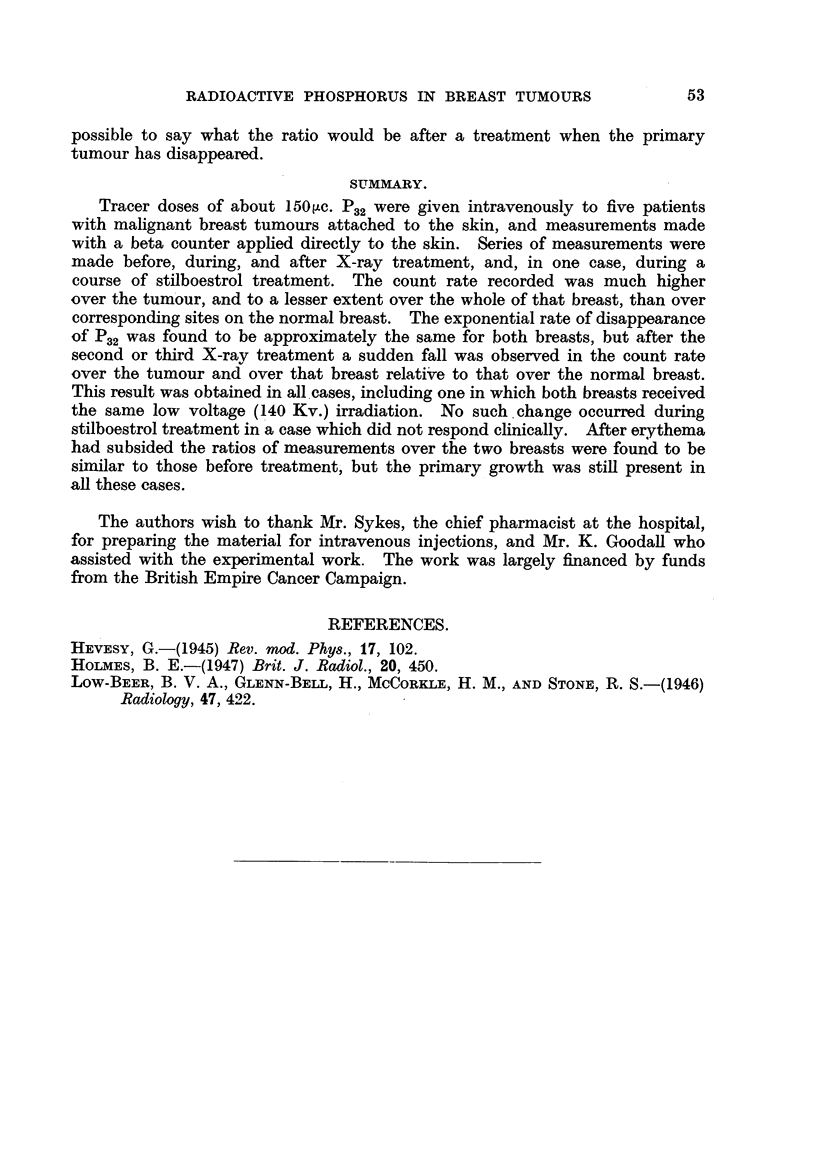# Investigation of the Effect of X-radiation on the Localization of Radioactive Phosphorus in Breast Tumours

**DOI:** 10.1038/bjc.1951.5

**Published:** 1951-03

**Authors:** F. Ellis, Cornelia Hoch-Ligeti, R. Oliver


### INVESTIGATION OF THE EFFECT OF X-RADIATION ON THE LOCALIZATION OF RADIOACTIVE PHOSPHORUS IN BREAST TUMOURS.

F. ELLIS, CORNELIA HOCH-LIGETI AND R. OLIVER.

*From the Radiotherapy and Cancer Research Departments, London Hospital, London, E.*1.

Received for publication January 25, 1951.

THE distribution of radioactive phosphorus can be investigated *in vivo* using a thin-walled G.M. counter tube. The maximum range for beta particles of $P_{32}$ in soft tissue is 8 mm., but the half-value thickness is only of the order of 0·6 to 0·8 mm. Consequently, measurements on the skin can only indicate the amount of phosphorus in the superficial layers of the body (effectively some 3 mm. deep). However, under favourable conditions, such measurements can be used to demonstrate the increased phosphorus up-take by malignant tumours. Low-Beer, Glenn-Bell, McCorkle and Stone (1946) have used this method with some success, as a diagnostic procedure, but its value is reduced by the low penetration of the beta rays and by the increased up-take also found in inflammatory conditions. The present series of experiments (started in November, 1948) was undertaken to investigate possible changes in the phosphorus content of malignant tumours during and after a course of X-ray treatment, and to attempt to correlate such changes with the reaction of the tumour to the irradiation.

### MATERIAL AND METHOD.

Only cases of mammary carcinoma with the tumour attached to the skin were selected ; the patients were in good general health.

On each occasion a tracer dose of about 150 $\mu$c. $P_{32}$ was given by intravenous injection in 5 c.c. phosphate buffer at pH 7·35. It has been found advisable to check, by counter measurements, that the radioactive material has actually left the site of injection, as, even after taking more than ordinary care, some unsatisfactory injections have been detected. This is particularly important, of course, when giving the isotope therapeutically, as dose calculations assume that the whole of the material enters the blood stream. No side effects of the injection were observed. Assuming uniform distribution, a body weight of 60 kg. and a biological half life of 8 days, the dose delivered was about 0·1 r in the first day and about 1·1 r over a period of some 4 weeks. The tumour may have received 4 to 8 times the dose calculated above, a total which is in excess of the generally agreed " tolerance " level. This was, however, of no consequence as the site was to receive an X-ray dose of 3000 to 4000 r.

A number of sites over the tumour, on the diseased breast, and at corresponding points on the normal breast, were marked with gentian violet, and measurements of the activities were made with a counter tube applied directly to the skin. A GM4 counter (General Electric Co., Ltd., England) was used, with conventional quenching and scaling circuits, the 0·001 inch thick duralumin window being covered with a sheet of cellophane to give some protection, and to avoid possible contamination of the window by the skin.

Some difficulty was experienced because of the rise of temperature of the counter in contact with the skin ; this led to a slight fall in count rate due to a shift of the counter voltage characteristic. In order to ensure that the operating point did not leave the plateau of the characteristic curve, the initial setting was some 150 volts above the critical voltage. The counter was checked against a standard beta ray source (uranium oxide with filter to remove alpha rays) before, and at intervals during the skin measurements, and the operating voltage adjusted slightly to maintain the same count rate. The background rate was checked before and after the series. In each case the complete set of measurements was repeated and a mean value for each site was used. It was not feasible to prolong the counting time at any site above about 3 minutes. As the count rates with this tracer dose were rather low, the measurements were usually limited to 1000 counts (3 per cent standard deviation) or, after several days and over normal tissue, to only 100 counts (10 per cent standard deviation). Consequently, the ratios of count rates over tumour and normal skin were not obtained very accurately, but the results sufficed to demonstrate large changes.

Series of measurements were made before, during, and 4 to 6 weeks after the treatment, at which time the X-ray erythema had subsided but the skin of the irradiated breast remained highly pigmented. Observations were made on alternate days during 2 to 3 weeks.

After intravenous injection, the radioactive phosphorus could be demonstrated immediately (5 to 8 seconds) in the tissues of the breast, but the concentrations continued to increase in both healthy and diseased breasts for 10 to 15 minutes. Considerably lower count rates were observed when the next series of measurements were made after an interval of 2 days, a change probably due to the excretion of $P_{32}$. Thereafter the fall was found to follow a roughly exponential form, corresponding to a " biological half-life " of between 7 and 9 days. Typical cases are illustrated in Fig. 1. The excretion of $P_{32}$ was not followed up with patients, but in preliminary experiments with rats it was found that, after intra-cardiac injection, a large percentage of $P_{32}$ was excreted in the urine in the first 48 hours. After that time, no $P_{32}$ could be demonstrated in the urine, but a small quantity continued to be found in the faeces over a period of at least 30 days.

The X-ray treatment was the same in Cases 1 to 4. Tumour dose of 3400 r (200 Kv., 1·0 mm., Cu. H.V.L.) was given (fractioned as 260 r on alternate days) using a glancing field technique. With Case 5 the whole chest wall received low voltage irradiation (140 Kv., 0·25 mm., Cu. H.V.L.) to a total of 2500 r in 5 weeks.

*Case records.*

(1) *Mrs. L. C—*, aged 57. Tumour in the left breast ; histologically solid trabecular, polygonal-celled carcinoma. The tumour was at least 15·0 cm. in diameter, hardest in the upper and outer quadrant. The skin was extensively involved. The mass was attached to muscle but not to the chest wall.

FIG. 1.—Fall of count rate over tumours in two typical cases as compared with physical decay.
(*a*) Physical decay.
(*b*) and (*c*) Count rates in two patients when not receiving X-ray treatment.

The actual count rates before and during the X-ray treatment are given in Table I. Apart from the breast measurements, readings taken over the forehead

TABLE I.—*Average Counts/Minute Above Background.*

| Site. | Before and during X-ray treatment. | | | | | | | | |
|---|---|---|---|---|---|---|---|---|---|
| Date | 18.ii. | 21.ii. | 23.ii. | 25.ii. | | 28.ii. | 2.iii. | 4.iii. | 7.iii. |
| Tumour | 801 | 260 | 240 | 197 | X-ray treatment started. | 118 | 86 | 61 | 42 |
| Tumour edge | 563 | 124 | 108 | 94 | | 66 | 50 | 45 | 36 |
| Diseased breast | 635 | 117 | 89 | 66 | | 53 | 51 | 41 | — |
| Normal breast | 359 | 87 | 63 | 77 | | 63 | 41 | 41 | 28 |
| Normal breast | 171 | 87 | 65 | 50 | | 41 | 48 | 42 | 33 |
| Axilla, tumour side | 500 | 122 | 149 | 102 | | 64 | 74 | 47 | 32 |
| Axilla, normal side | 430 | 159 | 149 | 98 | | 80 | 76 | 36 | — |
| Forehead | 444 | 324 | 275 | 200 | | 162 | 147 | 118 | 109 |

and over both axillae are also included. It is of interest to note that the readings over the forehead (indicating the $P_{32}$ content of the frontal bone) increased to the level found over the tumour and then decreased more slowly than the latter. Values of the ratio of count rates over the diseased breast to those over the healthy breast are presented in Fig. 2*a* and 2*b*. Before treatment the ratio of the count rates remained constant, but a break in the curves was observed 3 days after the irradiations started. The measurements in Fig. 2*b* were made 6 weeks after the end of the X-ray treatment, when the whole surface of the irradiated breast had a higher $P_{32}$ content than the healthy breast, the ratio between the two being similar to that before treatment.

The patient's condition deteriorated, and 7 months after treatment she died with extensive ulcerative carcinoma.

FIG. 2.—Ratio of count rate over diseased to that over normal breast.
*Mrs. L. C—* :

—— × —— × —— × —— Ratios over tumour.
—— ● —— ● —— ● —— Ratios over tumour periphery.
—— + —— + —— + —— Ratios over apparently normal tissue on diseased breast.

(*a*) Before and during X-ray treatment. Arrow marks first treatment.
(*b*) Six weeks after X-ray treatment.
The curve is shown dotted between the first two measurements after the injection of $P_{32}$, during which time the ratios were not steady.

(2) *Mrs. A. W—*, aged 60. Polygonal-celled carcinoma in the right breast. The tumour measured 11 × 9 × 6 cm. No palpable axillary glands.

In this, our first exploratory case, X-ray treatment started on the day following the injection of a tracer dose of $P_{32}$. The results are given in Fig. 3*a*. A sudden drop in the ratio occurred after the third irradiation. Four weeks after the end of the treatment a second tracer dose was given, and the results obtained are shown in Fig. 3*b*. For the first three readings the skin was still very red. By the time the erythema had subsided the count rates were rather low, but they appeared to be higher than normal over the whole of the diseased breast, without any localized area of high activity.

Two months after the irradiation total mastectomy was carried out. The patient is in good general condition with no evidence of recurrence 2½ years after the treatment.

(3) *Mrs. F. N—*, aged 65. Patient had a large hard right breast, with peau d'orange, containing a tumour, with ulceration and infiltration of the skin. There was pleural effusion on the left side.

FIG. 3.—Ratio of count rate over diseased to that over normal breast.
*Mrs. A. W—* :

—— × —— × —— × —— Ratios over tumour.
—— ● —— ● —— ● —— Ratios over tumour periphery.
—— + —— + —— + —— Ratios over apparently normal tissue on diseased breast.

(*a*) During X-ray treatment. Arrow marks first treatment.
(*b*) Four weeks after X-ray treatment. Erythema still present.
The curve is shown dotted between the first two measurements after the injection of $P_{32}$, during which time the ratios were not steady.

The measurements with this patient are of particular interest as she received a course of stilboestrol before starting X-ray treatment. As shown in Fig. 4*a* the ratio of count rates over the two breasts remained constant (or even tended to rise) during the stilboestrol treatment, which produced little clinical improvement. Five weeks after the stilboestrol treatment was finished, X-ray treatment started. After two X-ray doses a sudden fall in the ratio was again observed (Fig. 4*b*), but a third series of measurements, 4 weeks after the completion of the X-ray treatment, indicated ratios as high as those obtained before treatment (Fig. 4*c*).

The patient did not improve considerably. One year after treatment she developed neurological symptoms and died.

(4) *Mrs. H—*, aged 72. Advanced carcinoma of the right breast, a diffuse thickening occupying most of the breast.

Fig. 5 shows the measurements over the diseased breast relative to those over the normal breast. The ratios, fairly constant before treatment, dropped suddenly after the second irradiation and continued to fall towards unity. Because of the age of this patient the injection of $P_{32}$ was not repeated after treatment.

(5) *Miss E. R—*, aged 61. Stage III tumour of left breast.

Although in this case the *whole* chest wall was irradiated, the ratio of count rates over the diseased and normal breasts showed a sudden drop after the second irradiation (Fig. 6), indicating a diminution of $P_{32}$ uptake in the affected as compared with the normal breast. Measurements were also made over an isolated skin nodule separated from the primary growth by 6 cm. of apparently normal skin. The initial count rate over this nodule was only slightly lower than that over the primary growth, and the exactly similar changes were observed for these two sites.

Fig. 4*a*.

Fig. 4*b*.

Fig. 4.—Ratio of count rate over diseased to that over normal breast.
*Mrs. F. N—* :

—— × —— × —— × —— Ratios over tumour.
—— ● —— ● —— ● —— Ratios over tumour periphery.
—— + —— + —— + —— Ratios over apparently normal tissue on diseased breast.

(*a*) During stilboestrol treatment.
(*b*) Before and during X-ray treatment. Arrow marks first X-ray treatment.
(*c*) Four weeks after X-ray treatment.

The curve is shown dotted between the first two measurements after the injection of $P_{32}$, during which time the ratios were not steady.

FIG. 4c.

FIG. 5.—Ratio of count rate over diseased to that over normal breast.
*Mrs. H— :*

—— × —— × —— × —— Ratios over tumour.
—— ● —— ● —— ● —— Ratios over tumour periphery.
—— + —— + —— + —— Ratios over apparently normal tissue on diseased breast.

Before and during X-ray treatment. Arrow marks first treatment.
The curve is shown dotted between the first two measurements after the injection of $P_{32}$, during which time the ratios were not steady.

### DISCUSSION.

Although the number of cases is very small, the results appear to be of sufficient interest to justify publication, particularly as the present series of experiments has had to be discontinued because the team has dispersed. At a later date the authors hope to continue the investigation, although working at different institutions.

The count rate due to $P_{32}$ was found to be very much higher (between 3 and 8 times) over malignant mammary tumours attached to the skin, than over the corresponding parts of the healthy breast. A higher count rate was also recorded

FIG. 6.—Ratio of count rate over diseased to that over normal breast.
*Miss E. R— :*

—— × —— × —— × —— Ratios over tumour.
—— ● —— ● —— ● —— Ratios over tumour periphery.
—— + —— + —— + —— Ratios over apparently normal tissue on diseased breast.
—— * —— * —— * —— Ratios over isolated skin nodule.

Before and during X-ray treatment. Arrow marks first treatment.

over parts of the diseased breast remote from the palpable tumour, but it is not possible to decide whether this is due to a greater tissue vascularity or to some microscopic or sub-microscopic spread of the tumour.

The exponential rate of disappearance of $P_{32}$ was found to be approximately the same for diseased and normal breasts, but in all cases receiving X-ray treatment, a sudden fall in count rate over the tumour and over that breast was observed after the second or third irradiation.

It appeared that this sudden fall occurred first over the peripheral parts of the tumour, a drop over the central part following after one or two further irradiations.

In experiments with rats, X-radiation produced a marked inhibition of $P_{32}$ uptake in the thyomonucleic acid fraction in tumours and other tissues with high mitotic activity (Hevesy, 1945 ; Holmes, 1947). Although a different technique was used, these findings may account for the results reported above. It is not possible to decide, from the present experiments, whether the changes observed during the course of X-ray treatment of patients are due to change in metabolism of the tumour cells or to vascular damage. However, it may be possible to decide this by suitably controlled experiments.

Count rates, much higher than normal, were observed over areas of X-ray erythema, and it is therefore important to postpone measurements after treatment until this reaction completely clears. The ratios of count rates over the two breasts were then found to be similar to those obtaining before X-ray treatment, but, as the primary growths were still present in all these cases, it is not possible to say what the ratio would be after a treatment when the primary tumour has disappeared.

SUMMARY.

Tracer doses of about 150$\mu$c. $P_{32}$ were given intravenously to five patients with malignant breast tumours attached to the skin, and measurements made with a beta counter applied directly to the skin. Series of measurements were made before, during, and after X-ray treatment, and, in one case, during a course of stilboestrol treatment. The count rate recorded was much higher over the tumour, and to a lesser extent over the whole of that breast, than over corresponding sites on the normal breast. The exponential rate of disappearance of $P_{32}$ was found to be approximately the same for both breasts, but after the second or third X-ray treatment a sudden fall was observed in the count rate over the tumour and over that breast relative to that over the normal breast. This result was obtained in all cases, including one in which both breasts received the same low voltage (140 Kv.) irradiation. No such change occurred during stilboestrol treatment in a case which did not respond clinically. After erythema had subsided the ratios of measurements over the two breasts were found to be similar to those before treatment, but the primary growth was still present in all these cases.

The authors wish to thank Mr. Sykes, the chief pharmacist at the hospital, for preparing the material for intravenous injections, and Mr. K. Goodall who assisted with the experimental work. The work was largely financed by funds from the British Empire Cancer Campaign.

### REFERENCES.

Hevesy, G.—(1945) *Rev. mod. Phys.*, **17**, 102.
Holmes, B. E.—(1947) *Brit. J. Radiol.*, **20**, 450.
Low-Beer, B. V. A., Glenn-Bell, H., McCorkle, H. M., and Stone, R. S.—(1946) *Radiology*, **47**, 422.